# Toward Informative Representations of Blood‐Based Infrared Spectra via Unsupervised Deep Learning

**DOI:** 10.1002/jbio.70011

**Published:** 2025-03-24

**Authors:** Corinna Wegner, Zita I. Zarandy, Nico Feiler, Lea Gigou, Timo Halenke, Niklas Leopold‐Kerschbaumer, Maik Krusche, Weronika Skibicka, Kosmas V. Kepesidis

**Affiliations:** ^1^ Chair of Experimental Physics—Laser Physics Ludwig‐Maximilians‐Universität München (LMU) Garching Germany; ^2^ Laboratory for Attosecond Physics Max Planck Institute of Quantum Optics (MPQ) Garching Germany; ^3^ Center for Molecular Fingerprinting (CMF) Budapest Hungary; ^4^ Faculty of Physics University of Warsaw Warsaw Poland

**Keywords:** biomarkers, deep learning, disease diagnostics, infrared spectroscopy, liquid biopsies

## Abstract

This study explores using unsupervised deep learning to find a low‐dimensional representation of infrared molecular fingerprints of human blood. We developed a fully convolutional denoising autoencoder to process Fourier transform infrared (FTIR) spectroscopy data, aiming to condense the spectra into a set of latent variables. By utilizing the autoencoder's bottleneck architecture and a custom loss function, we effectively reduced noise while retaining essential molecular information. This method improved lung cancer detection accuracy by 2.6 percentage points in a case–control study. The resulting latent space not only compacts spectral data, but also highlights variables linked to disease presence, offering potential for improving diagnostics.

**Trial Registration:** German Clinical Trials Register (DRKS): DRKS00013217

## Graphical abstract

This study employs an unsupervised deep learning approach using a denoising autoencoder to distill blood‐based FTIR spectra into latent variables. By reducing noise and preserving essential information, the method enhanced lung cancer detection accuracy by 2.6%, providing a concise, clinically relevant representation of infrared spectra for improved diagnostics.

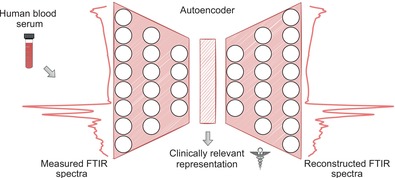

## 1 Introduction

Vibrational spectroscopy, utilizing Raman [1] or infrared techniques [2], has emerged as a potent analytical tool in medical research, promising rapid, noninvasive disease diagnosis [2, 3, 4]. Through classifying bacteria and cell (sub)types [5], distinguishing between benign and malignant tissues [6], and capturing the fingerprint spectra of biofluids [7, 8], this approach has demonstrated potential for contributing to advances in disease detection. Fourier transform infrared (FTIR) spectroscopy is widely used for such purposes. It provides unique insights into the molecular composition of biological samples by detecting the absorption of infrared light at various frequencies [9]. Thus, it proves versatile in analyzing diverse biomolecules, including proteins, lipids, and carbohydrates, facilitating disease detection [2, 3, 5, 7, 8, 10, 11, 12, 13, 14, 15, 16, 17, 18, 19, 20]. However, practical FTIR spectroscopy applications in medical diagnostics often face challenges related to systematic noise. Stemming from various sources such as sample collection, sample handling, instrument specifications, or drifts [21], noise can obscure spectral features critical for accurate diagnosis.

Deep learning has emerged as a potent tool in processing measurements across various scientific domains [22, 23, 24], leveraging its adaptability and generalization capabilities across different measurement types. These models excel in extracting meaningful patterns, features, and relationships from raw measurement data, making them suitable for the task of preprocessing. They were demonstrated to enhance data quality and relevance in scientific research [25, 26], medical imaging [27, 28, 29], industrial monitoring [30], and beyond. Convolutional neural networks (CNNs), a specific class of deep learning algorithms, excel in learning hierarchical representations from complex data. They were proved effective in handling diverse and high‐dimensional measurement datasets, particularly in medical applications [31, 32]. In various domains such as image processing [33], signal denoising [25], and feature extraction [34], CNN models effectively filter white noise [35], detect subtle patterns [36] and highlight crucial information [37]. Thereby, they contribute to improved accuracy and efficiency in subsequent analyses.

While deep learning models thrive when large datasets are available for training, they may struggle to generalize with restricted sample sizes. In this scenario, they risk overfitting by memorizing the idiosyncrasies of small datasets rather than capturing meaningful patterns. Obtaining sufficiently large clinical datasets for deep learning is challenging due to limitations in clinical studies. However, data augmentation techniques can offer a solution. One such approach in FTIR data analysis involves generating synthetic data by sampling from a multivariate Gaussian model fitted to the original measurement data.

Autoencoders are a class of deep neural networks designed for unsupervised learning and data compression [38, 39]. They consist of an encoder and a decoder module. The primary objective of autoencoders is to reconstruct the input data at the output layer while obtaining a condensed, information‐rich representation in the process. The encoder compresses the input data into a lower‐dimensional latent space, often referred to as the bottleneck or encoding. This bottleneck layer imposes dimensionality reduction, which constrains the information retained, typically resulting in a latent representation with reduced noise compared to the input. The decoder then reconstructs the original input from this compressed representation. By introducing constraints, such as prioritizing specific regions of the input during reconstruction via the loss function, the model is encouraged to retain crucial information. Autoencoders have their roots in denoising feature spaces [40] and have been utilized in classifying infrared spectra [41]. They are widely used in data denoising, dimensionality reduction, and feature learning [42, 43], making them well‐suited for identifying a latent representation of infrared measurements from liquid biopsies. This latent representation of spectral data can be used independently for further analysis, similar to traditional disease biomarkers.

This work introduces a fully convolutional denoising autoencoder designed to process blood‐based FTIR spectra. The training objective leverages domain knowledge about noise‐prone spectra regions, enabling targeted noise reduction in biologically relevant areas. Unlike traditional preprocessing methods, which often depend on heuristic noise reduction techniques [44], our unsupervised autoencoder autonomously learns to identify and eliminate noise directly from the spectra without requiring labeled data or repeated quality control samples under varying laboratory conditions. This results in an improvement in the quality of FTIR spectra. The impact of our research extends beyond mere noise reduction. We demonstrate the effectiveness of our autoencoder through a case–control study aimed at detecting lung cancer, a condition where early diagnosis is crucial for improving patient outcomes. Our findings indicate that the denoising autoencoder achieves classification results comparable to those obtained with conventional preprocessing methods [7, 8]. The key advantage, however, lies in the compressed latent space representation generated by the autoencoder, which captures the most informative aspects of the spectra. By selecting features from this latent space, we can enhance classification performance and improve disease detection. Additionally, we show that these features are correlated with the presence and progression of lung cancer and have the potential to improve upon the performance of established disease biomarkers. These variables, which can be extracted from measured spectra in a label‐free manner, hold promise as IR‐based biomarkers for clinical use.

## 2 Methods

### 2.1 Study Cohort and Measurements

The study design, statistical matching, sample collection, handling, FTIR measurements, and classical methods for spectral preprocessing have been extensively documented in previous studies [7, 8]. This study's cohort substantially overlaps with a previously assembled one utilized in research focused on lung cancer detection from blood serum [7]. To bolster statistical robustness, blood serum samples newly acquired within the same study from distinct individuals were incorporated, thus augmenting the sample size compared to the previous study.

All participants provided written informed consent under research study protocols regulated by the Ethics Committee of the Ludwig‐Maximilian‐University (LMU) of Munich (#17‐141, #17‐182). Adherence to pertinent ethical regulations, including Good Clinical Practice (ICH‐GCP) and the principles of the Declaration of Helsinki, was strictly observed throughout the study. Registration of the clinical trial (ID DRKS00013217) was completed in the German Clinical Trials Register (DRKS).

In total, spectra from 523 lung cancer patients and an equivalent number of matched asymptomatic control individuals were incorporated into the analysis. Controls were matched to cases based on age and sex through optimal full matching. Characteristics of the cohorts, in terms of the control covariates, are presented in Table 1.

**TABLE 1:** Characteristics of the cohorts.

Cancer	# Patients	Age (years)	% Female	BMI (kg/m^2^)
Yes	523	65 ± 10	46	26 ± 5
No	523	65 ± 10	46	26 ± 5

Of the 523 lung cancer patients, staging information based on the TNM Classification of Malignant Tumors (Union for International Cancer Control, UICC) is available for a subset of 238 patients. This information is summarized in Table 4 and utilized in the analysis described in Section 3.5.

### 2.2 Manual Preprocessing

Our spectral data underwent manual preprocessing techniques established in previous works [7, 8, 21]. Firstly, all spectra were truncated to the range of 1000–3000 cm^−1^. Subsequently, all spectra were normalized using the Euclidean (L2) norm. These preprocessing steps standardized the data across samples, ensuring consistency and removing common sources of variability, thus laying the groundwork for accurate classification results.

### 2.3 Simulated FTIR Spectra

To obtain sufficient data to train the autoencoder, we sampled from a multivariate Gaussian model fitted on real measurements within the train set. Using this model, 5000 unlabeled synthetic spectra that are utilized to train the autoencoder network are sampled. Before inserting the spectra into the autoencoder, vector normalization under the Euclidean norm was performed [7, 21]. A comparison between real and synthetic spectra is presented in the Figure S1.

### 2.4 Deep Learning Workflow

**FIGURE 1:**
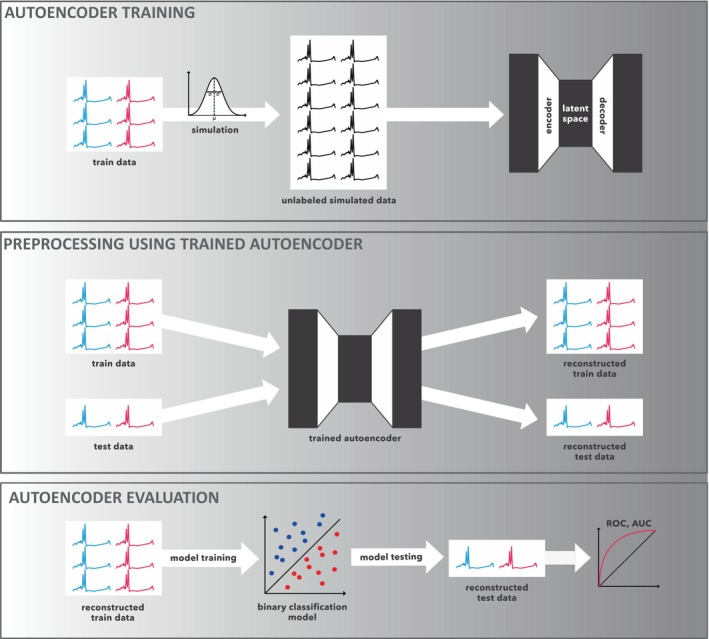
Workflow diagram showing training and evaluation of the autoencoder model in the case–control setting. As a first step, the cohort is randomly split into train and test sets. Then, synthetic train samples are generated from a multivariate statistical model fitted to the train set. These synthetic spectra are used to train the autoencoder. The trained autoencoder is then utilized for the preprocessing of all real data (train and test set). Finally, a binary classifier is trained on the reconstructed train data and applied to the reconstructed test data, where its performance is evaluated. This procedure is repeated 10 times, from the initial train‐test split.

Figure 1 illustrates the complete workflow for training and applying the proposed fully convolutional autoencoder model to a case–control study focused on lung cancer detection. The process begins by randomly splitting the dataset into training (90%) and testing (10%) subsets. Only the training set is used to generate synthetic data by sampling from a multivariate Gaussian model fitted on the real measurements. A total of 5000 synthetic FTIR spectra are produced to train the autoencoder, ensuring robust learning across diverse spectral variations. After the autoencoder is trained, it is employed to preprocess the real data, including both the training and testing sets. The denoised and preprocessed data is then used to train a binary classifier on the real, preprocessed training set. This classifier is designed to distinguish between lung cancer cases and controls, and its performance is subsequently evaluated on the preprocessed testing set. To ensure a more comprehensive and reliable assessment of the model's performance, we employ 10‐fold cross‐validation. Within this framework, the entire workflow (from data splitting, synthetic sample generation, and autoencoder training, to classifier evaluation) is repeated across 10 distinct folds. This results in 10 individually trained autoencoders, each optimized for a different fold of the data. By evaluating classification performance for each fold, we gain a robust understanding of how well the model generalizes to unseen data. This cross‐validation strategy minimizes the risk of overfitting, ensures the autoencoder learns meaningful noise reduction patterns, and provides a more reliable estimate of the model's classification accuracy and overall performance in the detection of lung cancer.

### 2.5 Neural Network Architecture and Training

**FIGURE 2:**
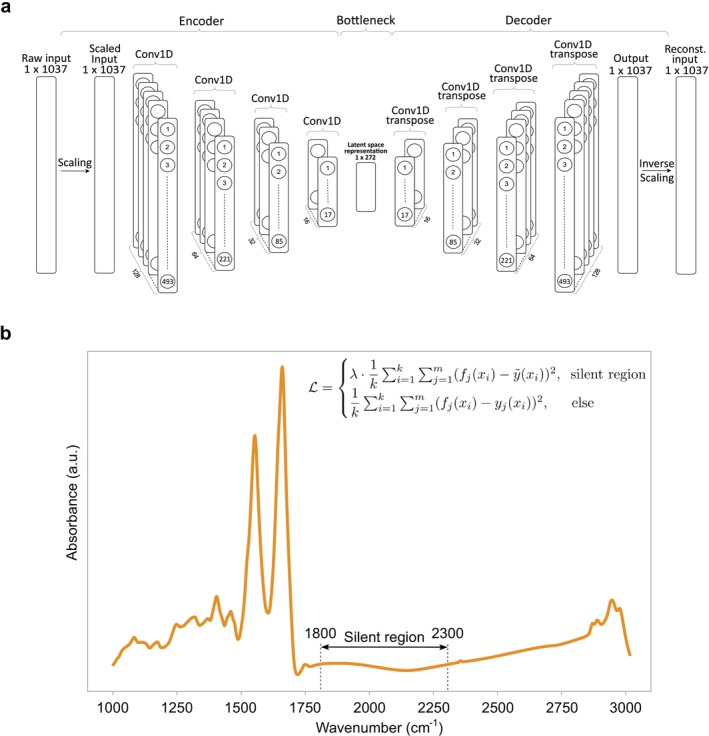
Autoencoder architecture and custom loss function. (a) Diagram illustrating the network architecture of the convolutional autoencoder. (b) Typical FTIR spectrum of blood serum in arbitrary units. The so‐called silent region, utilized for systematic noise removal by the autoencoder, is indicated between 1800 and 2300 cm^−1^. In this wavenumber range, there is minimal absorption, resulting in a relatively flat baseline. While the rest of the spectrum contains information about the molecular composition of the blood sample, the silent region is less informative regarding molecular composition. Thus, it is the optimal range for identifying various types of measurement drifts. The inset equation displays the two‐component custom loss function (mean squared error).

**FIGURE 3:**
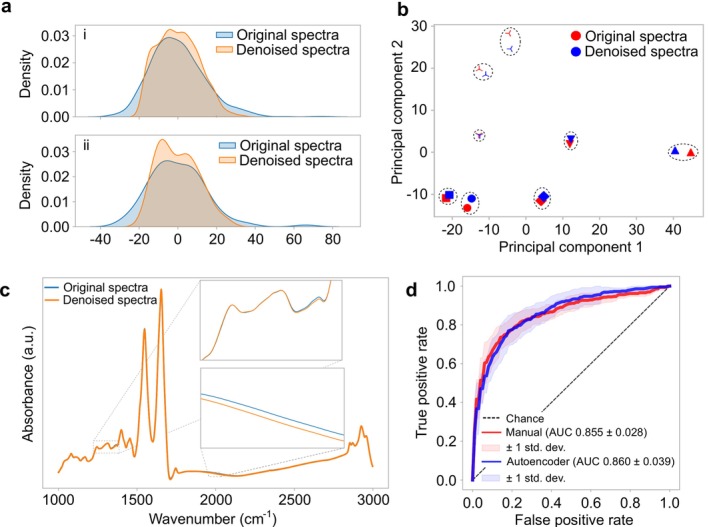
Denoising of infrared spectra. (a) Density plots of the first principal component from the silent region, for both raw and denoised spectra. Sub‐panel (i) corresponds to the analysis of the training data set, while sub‐panel (ii) corresponds to the same analysis of the test set. (b) Scatter plot of randomly selected measured spectra in the sub‐space of the first two principal components. (c) Example of a real raw measurement and its reconstructed spectrum. (d) Aggregated ROC curves for a binary classification model based on a logistic regression algorithm evaluated with 10‐fold cross‐validation.

Hyperparameter optimization of the autoencoder was performed on synthetic samples generated via sampling from a multivariate Gaussian model fitted on real measurements. To exploit symmetries and correlations that exist in the spectroscopic data, we subsequently implemented a fully convolutional autoencoder trained using mean squared error (MSE) as a loss function. This approach yielded results worse than the manual preprocessing. For this reason, we used the piece‐wise loss function described in the next subsection, which was specifically designed to address sources of variability observed in the silent region. In this case, slightly increased results were obtained (see Section 3). Hyperparameters of the fully convolutional autoencoder, such as the number of convolutional layers, filters per layer, activation functions, and learning rates, were tuned to enhance the model's capacity to capture essential features and patterns within the data. This capacity was assessed in a two‐fold manner: (i) by ensuring a reduction in variability in the silent region verified through density plots of the first principal component, as illustrated in Figure 3a, (ii) by ensuring that pairs of raw and reconstructed spectra were preserved, as depicted in Figure 3b. This procedure led to the proposed autoencoder architecture illustrated in Figure 2a.

We chose to use a CNN because it performed slightly better than a fully connected neural network of comparable size (number of layers) while having many fewer parameters. The architecture consists of a series of 1D convolutional [45] and transposed convolutional layers with the exponential linear unit (ELU) as an activation function [46]. The optimized hyperparameter settings are summarized in Table 2. Training of the autoencoder was performed using Keras (v.2.9.0) [47]. ADAM was selected as an optimizer for gradient descent with an overall learning rate of 0.001 [46]. Furthermore, early stopping [39] was used to ensure that training stops when an optimum for the loss function is found by recognizing stagnating learning. Besides, the models were chosen to be trained for 50 epochs by default with a batch size of 128. Ten percent of the synthetic training set was used for validation.

**TABLE 2:** Hyperparameters of the autoencoder.

Layer type	Num filters	Output shape	Num params
InputLayer	None	(1037, 1)	0
Conv1D	128	(493, 128)	6912
Conv1D	64	(221, 64)	434 240
Conv1D	32	(85, 32)	108 576
Conv1D	16	(17, 16)	27 152
Conv1DTranspose	32	(85, 32)	27 168
Conv1DTranspose	64	(221, 64)	108 608
Conv1DTranspose	128	(493, 128)	434 304
Conv1DTranspose	1	(1037, 1)	6785

Architecture characteristics	Hyperparameters
Activation	“elu”
Filter size	53
Strides	2
Optimizing strategy	Adam
Learning rate	0.001
Batch size	128
Validation split	0.1
Number of epochs	50
Total params	1 153 745

### 2.6 Custom Loss Function

Utilizing this autoencoder, our objective is to minimize measurement noise from FTIR spectra while retaining essential information regarding the individual biological variation of the samples. We introduce a custom loss function based on the MSE encoding domain knowledge to achieve this. FTIR spectra of blood serum exhibit a natural division into two regions: the “silent region” characterized by elevated water absorbance (within 1800–2300 cm^−1^), and the remainder, containing vital biological information about the sample being measured. Our tailored loss function operates distinctly within these regions, compelling the model to accurately reproduce spectra in the biologically relevant region while simultaneously mapping the silent region to a fixed spectrum (see Figure 2b). The utilization of a fixed spectrum as a reference promotes a more consistent and robust representation of the original measurement within this particular region, thereby minimizing system‐specific confounding factors that could hinder the performance of subsequent classification models.

In our approach, the average of a control sample (pooled human serum, BioWest, Nuaillé, France), repeatedly measured under varying laboratory conditions throughout the entire measurement campaign, serves as the fixed spectrum. However, averaging over all real biological samples could present an equally reasonable alternative. During training under this constraint, the autoencoder adjusts its weights to establish a transformation procedure that effectively addresses sources of variability observed in the training set, particularly patterns evident in the silent region. These variations may stem from instrument‐specific noise and drift, and discrepancies in sample handling.

For network output f, fixed spectrum $\tilde{y}$, and target spectrum y, the piece‐wise custom loss function is defined as follows,

(1) $$L=\left\{\begin{array}{ll} \;\lambda \cdot \frac{1}{k}\sum_{i=1}^{k}\sum_{j=1}^{m}{\left(f_{j}\left(x_{i}\right)-\tilde{y}\right)}^{2}, & \text{silent}\;\text{region}\\ \frac{1}{k}\sum_{i=1}^{k}\sum_{j=1}^{m}{\left(f_{j}\left(x_{i}\right)-y_{j}\left(x_{i}\right)\right)}^{2}, & \text{else} \end{array}\right.$$

The summation limits m and k correspond to the total number of spectra and wavenumbers, respectively, while λ is a hyperparameter controlling the relative importance between the two components of the loss function. This parameter allows us to adjust the importance of the loss function in the silent region relative to the loss originating from the rest of the spectrum. Given that the silent region encompasses less spectral coverage compared to the remainder of the spectrum, these two contributions differ in magnitude. Therefore, precise tuning of λ is necessary to accurately map the silent region to the desired outcome. Out of the range of tested amplification parameters spanning from one to 100 000, λ = 1000 yielded the most favorable results in terms of spectral reconstruction and noise reduction (see Section 3). The optimal parameter value was determined in the same way as the network's hyperparameters (see Section 2.5).

### 2.7 Binary Classification and ROC Analysis

To construct classification models, we utilized Scikit‐Learn (v.1.0.2) [48], an open‐source machine‐learning framework implemented in Python (v.3.9.13). Logistic regression served as the algorithm for binary classification, and the model parameters were determined using grid search. Model performance was evaluated using the receiver operating characteristic (ROC) curve, with the area under the curve (AUC) serving as a summary metric.

To rigorously account for the influence of disease progression on the ROC curve, we conducted binormal ROC analysis in addition to the empirical investigations. The binormal ROC curve is a classical model of ROC curves, allowing the finding of functional forms of empirical curves through model fitting. This enables one to investigate the influence of covariates on the ROC curve through ROC‐GLM regression. Within this framework, the ROC curve is modeled as a function of covariates using a generalized linear model (GLM). The ROC‐GLM regression model reads as follows.

(2) $$\Phi^{-1}\left(\mathrm{ROC}\left(t\right)\right)=h\left(t\right)+\beta_{0}X+\beta_{1}X\Phi^{-1}\left(t\right)$$

In the above equation, $h\left(t\right)$ is a monotonic function on $\left(0,1\right)$, $t\in \left(0,1\right)$ corresponds to the false positive rate, and $\Phi^{-1}\left(t\right)$ denotes the so‐called probit link, with Φ being the cumulative normal distribution function. The variable X corresponds to a covariate such as age, sex, BMI, or the stage of the disease. The coefficient $\beta_{0}$ specifies how the covariate influences the intercept of the ROC curve while $\beta_{1}$ describes its effect on the slope. Further details on the approach can be found in [49]. This analysis was performed in Stata (v. 17.0) utilizing the st0155 package [50]. GLMs were fitted utilizing a semiparametric approach. Parameter estimates for the covariates' influence on intercept and slope were assessed via bootstrapping (1000 bootstrap samples drawn separately from cases and controls). The significance of these estimates was determined using *p‐*values calculated from Wald statistics [50].

### 2.8 Latent Space and Feature Selection

To assess the predictive performance based on latent space features, the samples were first processed through the encoder of the trained network, generating latent space representations for each 10‐fold cross‐validation. Sequential feature selection, implemented using Scikit‐Learn [48], was then applied to determine the optimal number of input variables for the binary classification algorithm. This process utilized a backward elimination approach with automatic feature selection and low tolerance settings, meaning features were iteratively removed until any further reduction significantly impaired model performance. This method allowed the model to retain only the most critical features, eliminating those that were redundant or less informative. Once the latent variables for each sample were extracted, binary classification was performed in the same manner as for the spectral data.

## 3 Results

### 3.1 Denoising Infrared Spectra

Figure 3 shows the effect of applying the trained autoencoder network on the real spectra of our cohort. Figure 3a depicts density plots of the first principal component of the silent region for both the original and denoised spectra. The reduction in variability is evident in both the train and test measurements, suggesting a noise reduction. However, since the variability is reduced, one needs to investigate how information from the medically relevant part of the spectrum is altered. To this end, Figure 3b shows a scatter plot of randomly selected measured spectra in the sub‐space on the first two principal components. In this plot, we depict both the original and reconstructed (via autoencoder) version of each selected spectrum. The two spectra corresponding to the same sample form distinct pairs. This means that the distance between reconstructed and original spectra, that is, the reconstruction error, is smaller than the distance between different samples. This indicates that the autoencoder keeps the biological variation intact to a great extent while reducing measurement noise. This implies that applying the autoencoder meets the main goal of preprocessing, that is, to increase the relevant signal‐to‐noise ratio. Figure 3c shows a randomly selected original raw measurement and its reconstructed version. This comparison demonstrates that reconstructed spectra preserve the characteristics of raw measurements and can be successfully used for further analysis. In Figure S2a,b, a more detailed comparison between the original and reconstructed spectra based on (i) the effect size and (ii) a *t*‐test can be found. Additionally, Figure S2c depicts the absolute value of the residual difference in absorbance between the original and reconstructed spectra for different samples of the train set. This figure reveals a very diverse set of transformations produced by the neural network, triggered by the variable noise patterns in each measurement.

### 3.2 Application to Lung Cancer Detection

After identifying signatures consistent with an increased signal‐to‐noise ratio, we evaluated the autoencoder's utility in distinguishing lung cancer patients from non‐symptomatic controls. To ensure robust and generalizable performance, we employed 10‐fold cross‐validation, testing the model across different subsets of the data. Specifically, the dataset, consisting of 523 cases and 523 controls, was split into training and testing sets 10 times, with each fold holding out a distinct subset for testing while using the remaining samples for training.

For the manual preprocessing approach, raw measurements were processed using the standard manual steps described in Section 2. These preprocessed training and testing sets were directly used for binary classification with a logistic regression model, and performance was evaluated on the held‐out test sets across the 10 folds.

In the autoencoder‐based approach, the training set in each fold was first augmented with 5000 synthetic spectra generated from the training samples. The autoencoder was trained on these synthetic samples to learn robust spectral representations. Once trained, the autoencoder was used to preprocess both the training and testing sets of real samples. The reconstructed spectra were then used to train a logistic regression model on the reconstructed training data, and performance was evaluated on the reconstructed test sets.

The ROC curve for each fold was calculated, and the test results were aggregated to determine overall performance, as shown in Figure 3d. This figure compares the two approaches: (i) manually preprocessed spectra and (ii) spectra reconstructed via the autoencoder. The manual preprocessing approach achieved an average AUC of 0.855 across the test sets. The autoencoder‐based approach slightly improved performance, achieving an average AUC of 0.860. Detailed results for individual folds are provided in Table S1.

### 3.3 Investigating the Latent Space

An autoencoder captures information by passing the data through an information bottleneck. This process converts data into a reduced representation known as code or latent space. The latent space consists of a set of high‐level variables that capture the essential information of the input data. These latent variables are used for the faithful (and denoised) reconstruction of the input data by the decoder part of the network, thus already containing all the necessary information from the original measurements. Instead of reconstructing the original spectrum using the full autoencoder, the trained encoder part can be used stand‐alone. This yields a compressed version of the spectrum; in our case, this is a set of 272 latent variables. In principle, one can train a classification model based on these variables, since they already encode all information needed for reconstructing the spectra.

As shown in Figure 1, for each cross‐validation fold, different training data was used, resulting in 10 different models. Due to the randomness in the initialization of weights and slight variations in training, there is no guarantee that the same latent variable will represent the same information across different folds. Thus, the features in the same position might capture different representations of the original measurement, meaning that the latent space features cannot be compared pairwise, only aggregated measures can be used to evaluate the overall performance.

**FIGURE 4:**
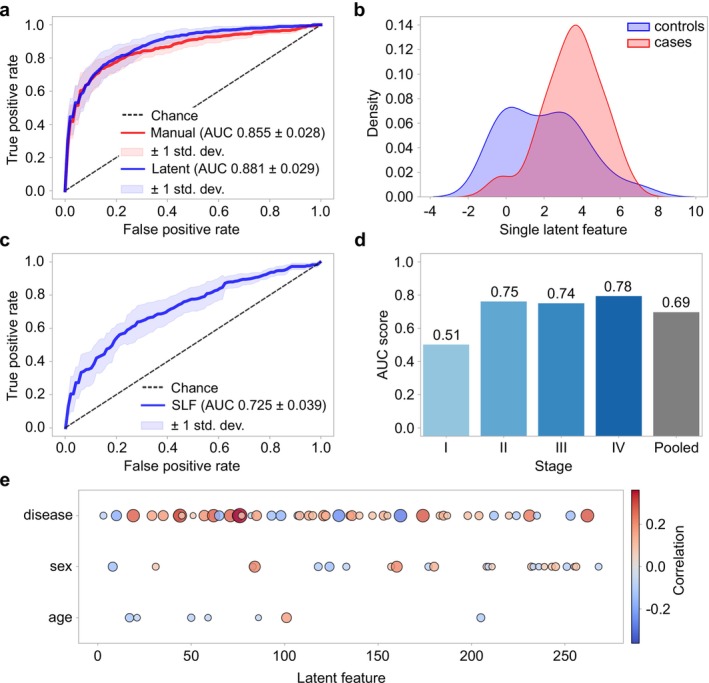
Latent space analysis. (a) ROC curves comparison for a binary classification model based on the latent features and the manual preprocessing. The evaluation was performed via 10‐fold cross‐validation, and the aggregated results are shown. (b) Density plots of the single latent variable characterized by the highest correlation with the disease status in fold four. The red curve corresponds to cases, and the blue curve to controls of the test set. (c) Aggregated ROC curve for a binary classification model based on the single latent feature (SLF). (d) Binormal ROC curves showing how the single latent feature's capacity to discriminate between cases and controls depends on the cancer stage. Evaluations are performed on the train set. (e) Correlation between the latent features and the disease status, subject's sex, and age. The latent space is reduced to those latent dimensions that are only correlated with one of the variables. The results shown here correspond to a randomly selected fold.

To evaluate the predictive performance using latent space features, the encoder was used to generate latent representations for all samples across the 10 folds. Next, backward feature selection was employed to identify the optimal set of input variables for the binary classification task. Once the final feature set was determined, logistic regression, combined with grid search for hyperparameter optimization, was used to assess the model's ability to detect lung cancer. As shown in Figure 4a, the aggregated AUC score from the 10‐fold cross‐validation was 0.881, representing an improvement of 2.6 percentage points over manual preprocessing methods. The detailed results for each fold are provided in Table S1. In addition, a stability analysis of the architecture is presented in Table S2.

Paired *t*‐tests were conducted to compare the predictive performance of three data processing approaches: manual preprocessing, autoencoder‐based reconstruction, and latent space features. The results revealed no statistically significant difference between manual preprocessing and autoencoder‐based reconstruction (*p* = 0.607). However, latent space features significantly outperformed both manual preprocessing (*p* = 0.016) and reconstructed signals (*p* = 0.015), highlighting their superior effectiveness in improving model performance for lung cancer detection. Among the three approaches, latent space features achieved the best performance, outperforming the use of reconstructed signals for classification. This result may be attributed to the curse of dimensionality—a phenomenon where the performance of certain machine‐learning algorithms deteriorates as the number of features or dimensions increases [51].

### 3.4 Latent Representation Analysis

We aimed to examine how information is stored in the latent space. Our prior studies show that molecular fingerprints are influenced by various factors, with demographic characteristics and diseases being the most significant [18]. To further investigate the information content of the latent features, the correlation with two basic demographic variables, age and sex, and disease status was measured. The correlations were calculated based on the train set using point‐biserial correlation in the case of binary features and Pearson correlation in the case of features with real values. Several latent variables demonstrate a particularly high correlation with the disease outcome in each fold, while the correlations with demographic features are also sufficiently strong. The average and standard deviation of the maximum absolute correlations for all three variables are shown in Table 3a. In Figure S3, the results for each 10 fold are visualized along with the cross‐correlations.

**TABLE 3:** Latent dimension maximum correlations.

	Disease	Sex	Age
(a) Overall maximum correlations			
Mean	0.393	0.275	0.234
Std	0.032	0.027	0.020
(b) Singular maximum correlation			
Mean	0.333	0.222	0.155
Std	0.029	0.017	0.020

**TABLE 4:** Characteristics of the cohort of lung cancer patients used in the evaluation shown in Figure 4d.

Stage	# Patients	Age (years)	% Female	BMI (kg/m^2^)
I	35	70 ± 9	46	26 ± 6
II	23	69 ± 9	26	25 ± 4
III	73	68 ± 10	34	25 ± 5
IV	107	68 ± 9	54	25 ± 5

**TABLE 5:** *p*‐values for comparing stage‐specific AUCs shown in Figure 4d.

Stage	I vs. II	I vs. III	I vs. IV	II vs. III	II vs. IV	III vs. IV
Intercept	0.004	0.001	0	0.589	0.931	0.427
Slope	0.394	0.647	0.57	0.249	0.222	0.951

After revealing that the latent space is relevantly correlated with all of our influencing factors, we aimed to analyze the partially disentangled representation of the latent space with features that are correlated with only one disease, age, and sex. This means that those latent features, which were correlated with two or more of the abovementioned influencing factors, were excluded. This reduced latent space still contains valuable features, which are strongly correlated with the three analyzed variables. The aggregated maximum values are summarized in Table 3b. The correlation of reduced latent space is visualized for a randomly selected fold in Figure 3e.

### 3.5 From Latent Variables to Disease Markers

Motivated by the amount of correlation, we opted to train a classification model based solely on the latent feature with the maximum correlation with the disease status for each fold. Figure 4b shows density plots of this single latent variable that correlates the strongest with the disease status for a randomly selected fold. The red curve corresponds to the cases and the blue curve to the controls of the test set. The exhibited behavior is similar to that of classification scores obtained by a binary classification model or a measured blood biomarker. This motivates the construction of a ROC curve based solely on this particular single latent feature (SLF). Figure 4c shows the aggregated ROC curve based on the SLF values.

The similarity between the single latent variable and a typical disease biomarker motivated us to apply a standard approach used in the field of medical statistics that allows us to investigate covariate effects on the performance of predictive biomarkers. This approach is known as ROC‐GLM regression modeling [49] and is described in the methods section. Using this method, one can evaluate the effect of a given covariate on the ROC curve of a binary classification model. Here, we apply this method to investigate the effect of disease progression, in terms of stage, as defined according to the TNM Classification of Malignant Tumors (Union for International Cancer Control (UICC)). Fitting the regression model to the data yields the intercept and slope coefficients with associated *p*‐values, which can be seen in Table 5. On the intercept *p*‐values, we can see that Stage I is significantly different from the other three stages. This aligns with prior findings that Stage I lung cancer cannot be detected using blood‐based FTIR spectroscopy, as demonstrated in a previous study [19]. In Figure 4d, we visualize this dependence of the SLF's AUC scores on the stage for a randomly selected fold.

### 3.6 Comparison to Classical Biomarkers

**FIGURE 5:**
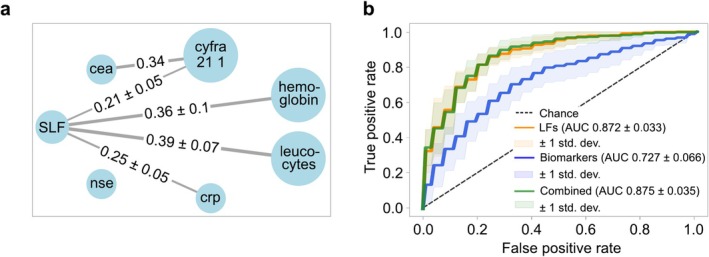
Biomarker analysis. (a) Network analysis showing correlations between the single latent variable and established markers for lung cancer. (b) Comparison of the already established lung cancer biomarkers with the latent features. The combined performance is also shown here.

Part of biomarker analysis is investigating the relationship to other biomarkers relevant to the same physiological condition. In Figure 5a, we show the result of network analysis based on Pearson correlation. We observe that in each fold the single latent variable correlates relatively strongly with four known markers for lung cancer, namely CRP, CYFRA 21 1, leukocytes, and hemoglobin [52]. This analysis suggests that extracted latent variables could be used as potential disease markers.

To compare the predictive performance of the latent variables with biological biomarkers, we used all 10 models to extract the latent representations. The biomarker data had several missing values, with NSE, CYFRA 21‐1, and CEA markers being particularly affected. These markers had over 50% missing data, primarily from the control group, where only 10% had available values for these parameters; hence, they were excluded from the analysis. The remaining three markers: hemoglobin, leukocytes, and CRP were included, though the control‐to‐case ratio was skewed at 1:2, with twice as many case samples as controls. This means the results are not directly comparable to previous analyses due to differences in the datasets. The results show that the latent features perform better than the biomarkers in predicting outcomes. It is safe to conclude that our latent features can enhance the classification performance of lung cancer subjects. The aggregated ROC curve can be seen in Figure 5b.

## 4 Conclusions

This study presents a novel approach for processing FTIR measurements from human blood, utilizing a fully convolutional denoising autoencoder based on unsupervised deep learning. Our approach not only achieves noise reduction but, more critically, uncovers a learned low‐dimensional representation of the measured spectra. The exploration of this latent space opens new opportunities for identifying infrared‐based disease markers that encapsulate medically relevant information in a compact set of variables. These variables can be leveraged for disease detection and characterization, as demonstrated in our findings, where even a single latent variable effectively functions as a classical biomarker. Unlike traditional biomarkers that reflect the abundance of a specific protein, these latent variables capture a broader spectrum of molecular information across the entire infrared range.

While the results are promising, it is crucial to acknowledge the limitations of our approach. A key challenge remains in ensuring that the autoencoder‐derived features can be generalized to external datasets, particularly in multicentric clinical studies that encompass diverse populations and varying measurement conditions. Without validation in such well‐designed studies, there is a risk of overfitting the training data, a common limitation of neural network‐based methods. This underscores the importance of rigorous external validation to establish the robustness and clinical utility of the proposed framework.

Looking forward, we aim to advance the autoencoder architecture to better extract disease‐specific latent features and improve performance. To enhance the generalizability of our framework, we plan to incorporate data from diverse studies spanning different populations and conducted with various measurement devices, thereby capturing broader variability in spectral data. Ongoing research includes incorporating additional loss functions in the latent space to enhance the correlation between latent variables and disease outcomes. Other possibilities include leveraging generative AI techniques, such as variational autoencoders, to learn disentangled and interpretable representations of the independent generative factors within the spectral data without supervision [53, 54].

Our research marks a significant step toward identifying informative representations of blood‐based infrared spectra. By harnessing deep learning, we contribute to the evolution of infrared spectroscopy as a powerful and transformative technology for medical diagnosis and patient care.

## Author Contributions

**Corinna Wegner and Zita I. Zarandy:** investigation; formal analysis; visualization; methodology; writing – original draft preparation; writing – review and editing. **Nico Feiler:** investigation; formal analysis; visualization; methodology; writing – review and editing. **Lea Gigou:** investigation; formal analysis; methodology; writing – review and editing. **Timo Halenke:** validation; writing – review and editing. **Niklas Leopold‐Kerschbaumer:** methodology; writing – review and editing. **Maik Krusche:** formal analysis; writing – review and editing. **Weronika Skibicka:** writing – review and editing. **Kosmas V. Kepesidis:** conceptualization; supervision; project administration; methodology; writing – original draft preparation; writing – review and editing.

## Conflicts of Interest

The authors declare no conflicts of interest.

## Supporting information


Data S1.

## Data Availability

The data that support the findings of this study are available on request from the corresponding author. The data are not publicly available due to privacy or ethical restrictions.
