# Supplementary material for: Toward Informative Representations of Blood‐Based Infrared Spectra via Unsupervised Deep Learning
Source: J Biophotonics. 2025 Mar 24;18(8):e70011. doi: 10.1002/jbio.70011 (PMC12318647; doi:10.1002/jbio.70011)
Supplement: Supplementary file 1 — Data S1. [file JBIO-18-e70011-s001.pdf]

Supplementary information for "Toward informative representations of blood-based infrared spectra via unsupervised deep learning"

Corinna Wegner[a†], Zita I. Zarandy[abc†], Nico Feiler[ab], Lea Gigou[abc], Timo Halenke[ab], Niklas Leopold-Kerschbaumer[ab], Maik Krusche[a], Weronika Skibicka[d], Kosmas V. Kepesidis[abc*]

Additional information about the individual cross-validation results, supplementary figures, and additional investigations are included herein to bolster and complement the primary text.

1 Individual fold results

In this appendix, we provide the detailed results for each fold of the cross-validation process conducted for all three lung cancer classification variants. Each fold was evaluated separately to assess the model's performance across different subsets of the data. The results are presented in terms of test set AUC scores in Table S1, providing a clear view of how well the model performed on each fold.

Table S1: Cross-validation AUC score results

# Fold	manual	autoencoder	latent
1	0.883	0.916	0.898
2	0.875	0.859	0.880
3	0.856	0.828	0.840
4	0.817	0.874	0.887
5	0.889	0.870	0.891
6	0.869	0.888	0.905
7	0.843	0.851	0.910
8	0.857	0.880	0.883
9	0.872	0.872	0.903
10	0.795	0.766	0.814
mean	0.855	0.860	0.881
std	0.028	0.039	0.029

2 Stability test of the architecture

To evaluate the stability and robustness of the autoencoder architecture, we performed a stability test on the first fold of our cross-validation. This test aims to determine whether the model consistently converges to a good solution despite different random initializations of the network weights. The autoencoder was trained 10 times on the first fold, each time with different random initial weights but using the same dataset and hyperparameters. The performance of the model was measured by the AUC score on the lung cancer test set for each run.

The results, summarized in Table S2, show that the model achieved consistent AUC scores across all 10 stability test runs, with scores ranging from 0.893 to 0.930. The mean AUC score was 0.913 with a standard deviation of 0.013, indicating a high level of consistency. This consistent performance suggests that the architecture is stable and reliably converges to an effective solution despite variations in initial conditions. The stability observed in this fold suggests that the architecture is robust to random initialization. This stability test provides initial evidence that the model can reliably achieve good performance when trained.

Table S2: Stability test AUC score results

Rerun	AUC Score
1	0.926
2	0.893
3	0.904
4	0.922
5	0.915
6	0.901
7	0.923
8	0.908
9	0.930
10	0.907
mean	0.913
std	0.013

3 Original and generated spectra comparison

To evaluate the similarity between real and generated spectra, Figure S1 presents analyses across multiple metrics. For an accurate comparison, 1046 generated spectra were compared to 1046 real samples. In subplot (a), the absorbance spectra of the original and generated samples demonstrate minimal deviations, highlighting accurate spectral generation. Here only the first 100 samples from each group are visualized. Subplot (b) displays the ROC curve for distinguishing real and generated spectra using Logistic Regression, with an AUC of 0.53, indicating that the generated spectra are nearly indistinguishable from the original. Lastly, subplot (c) compares the absorbance values at the wavenumber corresponding to the maximum absorbance value. The strong overlap between

[†] *Contributed equally to this work*

[*] *Corresponding author: kosmas.kepesidis@lmu.de*

[a] *Ludwig-Maximilians-Universität München (LMU), Chair of Experimental Physics - Laser Physics, Garching, Germany*

[b] *Max Planck Institute of Quantum Optics (MPQ), Laboratory for Attosecond Physics, Garching, Germany*

[c] *Center for Molecular Fingerprinting (CMF), Budapest, Hungary*

[d] *University of Warsaw, Faculty of Physics, Warsaw, Poland*

real and generated spectra further confirms the stability and accuracy of the generation.

4 Original and reconstructed comparison

To compare the means of the original and the reconstructed spectra the effect size was calculated and a two-sample t-test was performed. Figure S2 presents these metrics across different wavenumbers. For easier understanding, the metrics were smoothed using a sliding window function to reduce noise and highlight underlying trends, visualized in red. On subplot (a) it can be seen that the effect size is considered very small in most wavenumbers, since it barely reaches 0.01. Subplot (b) shows that the p-values of the two-sample t-test are mostly in the insignificant range, meaning that there is no statistically significant difference between the means of the two groups of spectra. The end of the silent region has significant differences according to the two-sampled t-test. This can be due to the custom loss function. This comparison suggests that the reconstructed spectra are highly similar to the original spectra, indicating successful and stable spectral reconstruction.

To visualize the differences indicated by the two metrics, we randomly chose six spectra and visualized the residual difference between the original and the reconstructed spectra. It can be seen in subplot (c). In each spectrum, a significant difference can be observed in the Amide I and II region and toward the end of the spectra, which corresponds to the results coming from the effect size comparison.

5 Latent space correlations

In addition to Table 3a, which presents the maximum correlation between latent variables and the three key influencing factors, we also analyzed the cross-correlation among them. The three latent variables were chosen based on their highest correlation with features: disease, sex, and age for each 10-fold individually.

Figure S3 presents the cross-correlation of the selected variables, highlighting the stability of relationships between latent variables and key factors across different cross-validation folds. Notably, the maximum correlations are consistently scaled across folds, with the disease variable consistently showing the strongest correlation with a latent variable. Additionally, the correlation with the sex feature is always equal to or greater than that with the age feature. However, the cross-correlations exhibit significant variation. Some latent variables show strong correlations with all three key factors, while others are particularly correlated with only one of these influencing features.

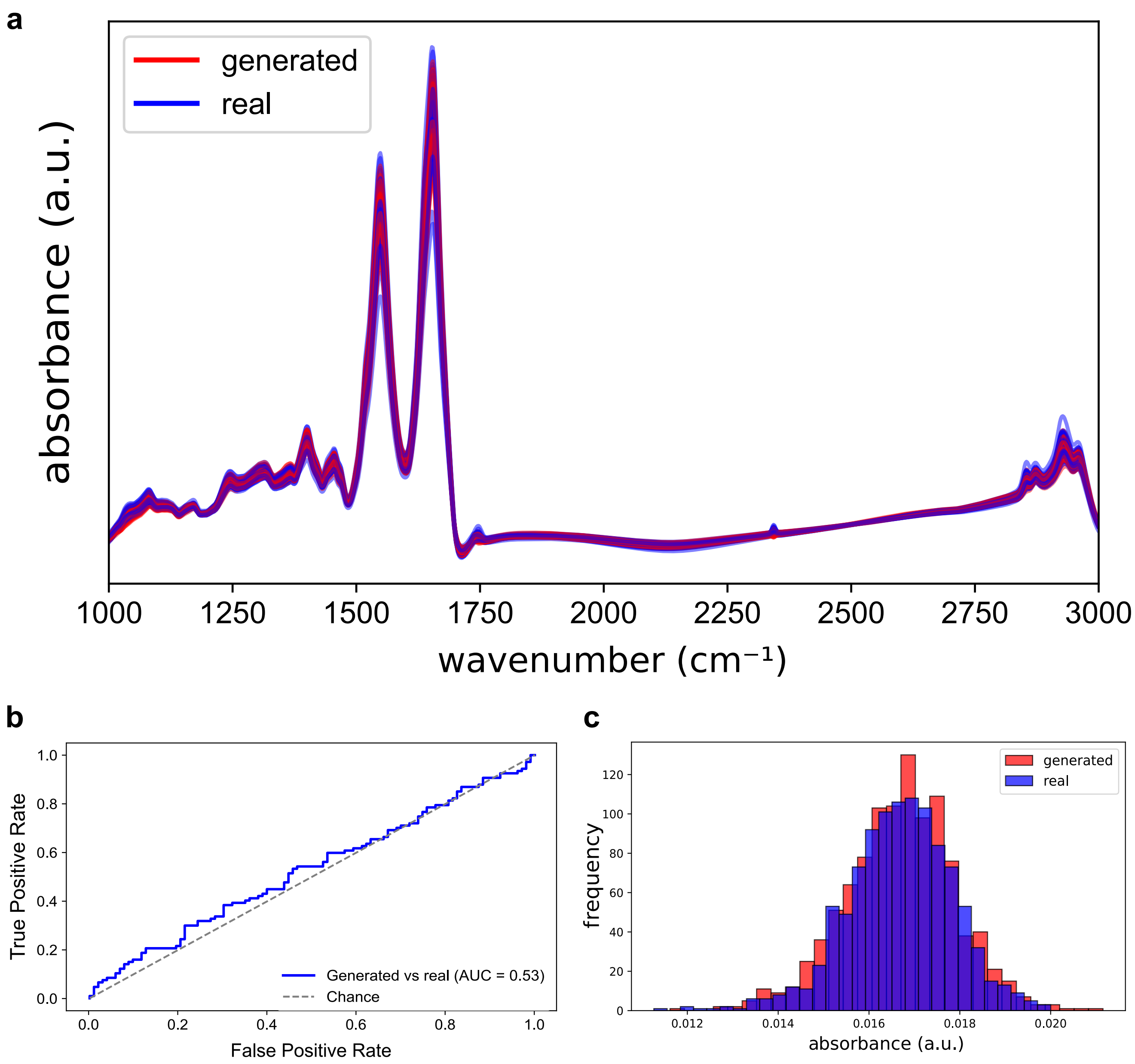

Figure S1: Comparison of original and generated spectra using multiple metrics. 1046 generated samples were compared to 1046 real samples. (a) The absorbance spectra of the original and generated samples, displaying the first 100 samples from each group. (b) The ROC curve for distinguishing real and generated spectra. (c) Absorbance distributions at the wavenumber corresponding to the maximum absorbance value, comparing the real and generated spectra.

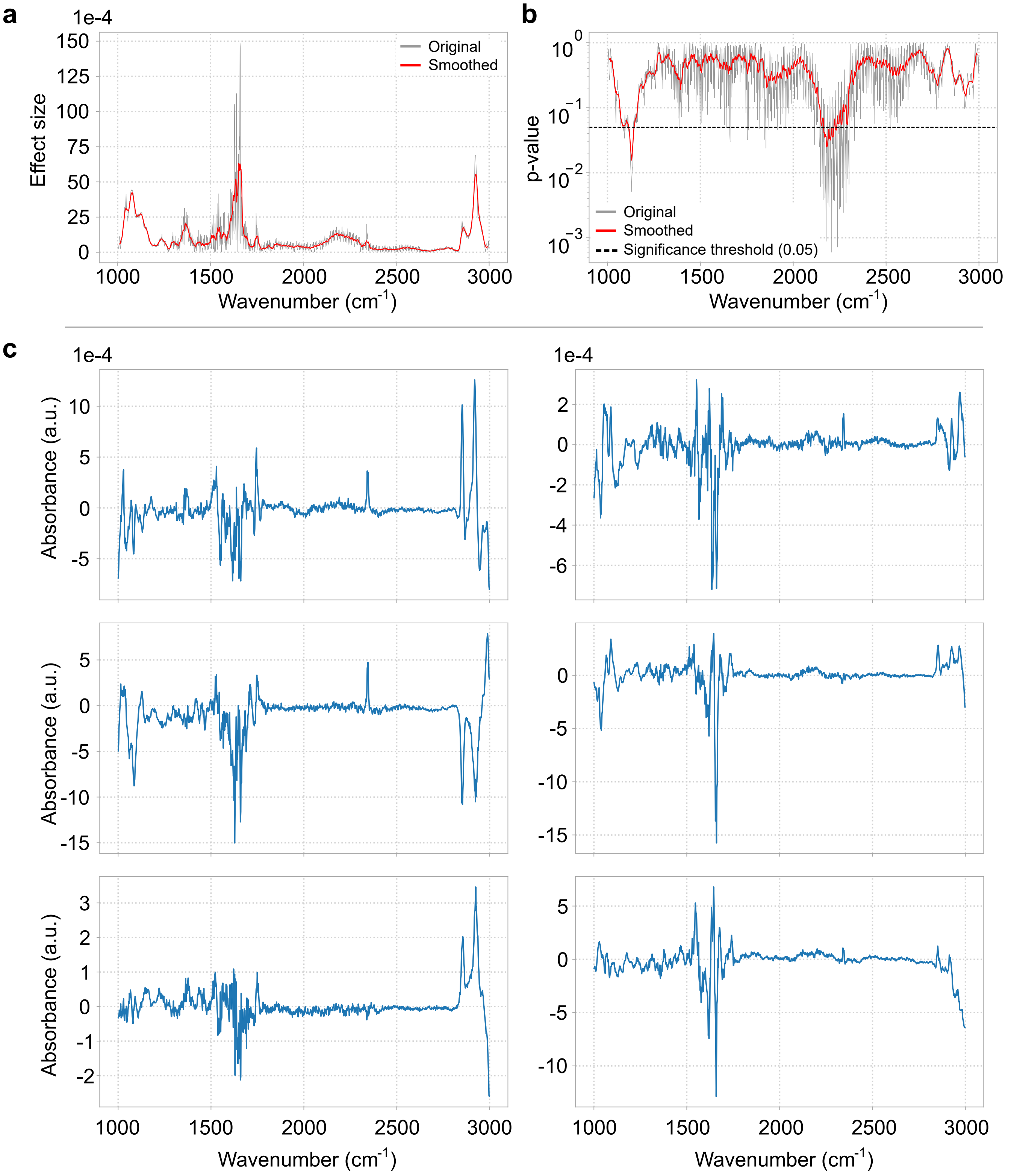

Figure S2: Original and reconstructed spectra comparison. The metrics were smoothed using a moving average sliding window with a window size of 10. (a) Effect size per wavenumber of real vs. reconstructed spectra. (b) Two-sampled t-test p-value per wavenumber of real vs. reconstructed spectra. (c) The residual difference in absorbance between original and reconstructed spectra for different samples in the train set.

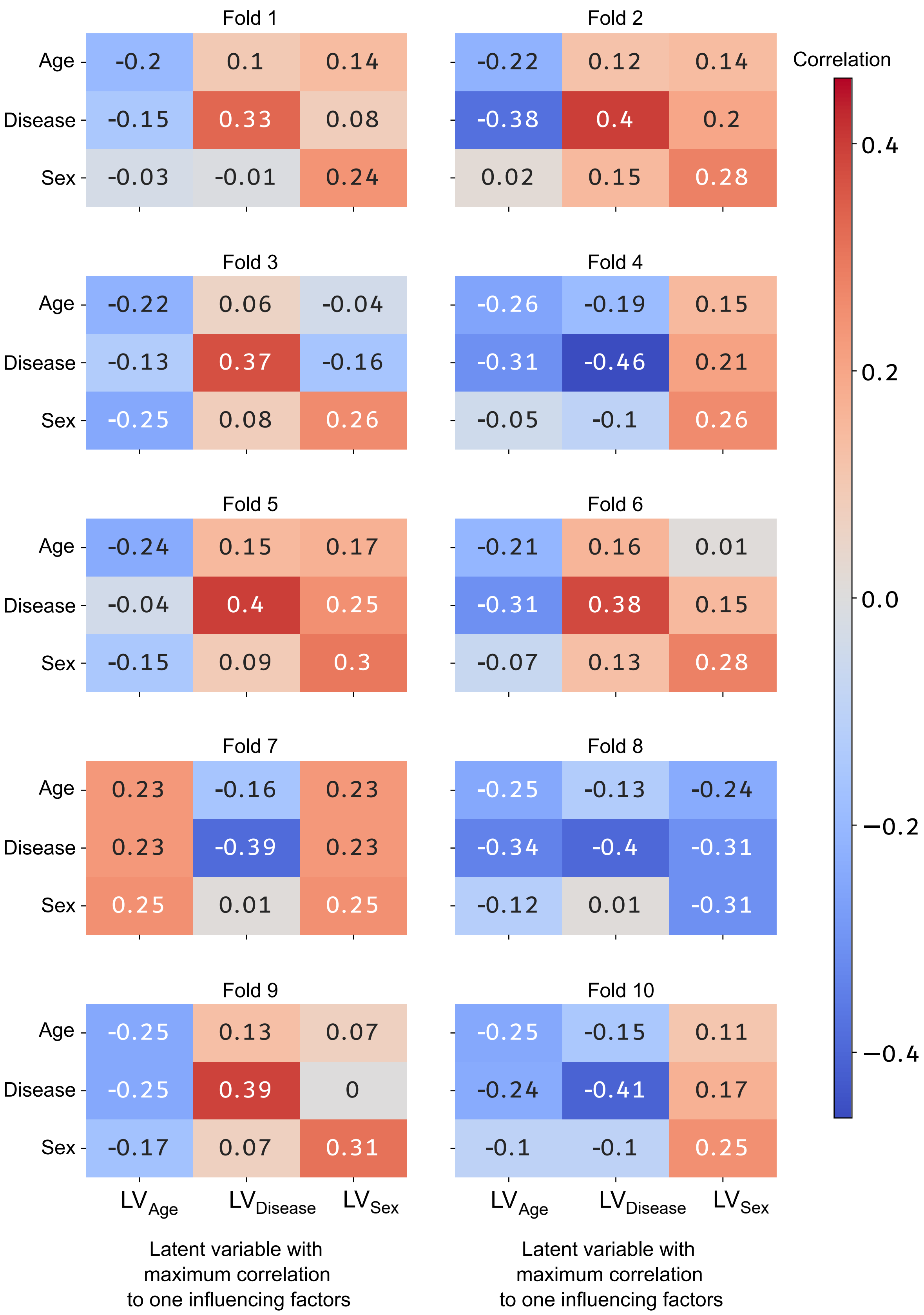

Figure S3: Correlation heatmaps of latent variables and spectra influencing features across 10 cross-validation folds. Each subplot corresponds to one of the 10 folds, displaying the correlation values between selected latent variables and the features. One latent variable with the maximum absolute correlation was selected for all three influencing factors.
